# Systematic review of cost and cost-effectiveness of different TB-screening strategies

**DOI:** 10.1186/1472-6963-11-247

**Published:** 2011-09-30

**Authors:** Albert Nienhaus, Anja Schablon, José Torres Costa, Roland Diel

**Affiliations:** 1 University Medical Center Hamburg-Eppendorf, Institute for Health Service Research in Dermatology and Nursing - Hamburg, Germany; 2 Occupational Health Division, S. João Hospital, EPE - Porto, Portugal; 3 Department of Pneumology, Medical School Hannover, Hannover, Germany

## Abstract

**Background:**

Interferon-γ release assays (IGRAs) for TB have the potential to replace the tuberculin skin test (TST) in screening for latent tuberculosis infection (LTBI). The higher per-test cost of IGRAs may be compensated for by lower post-screening costs (medical attention, chest x-rays and chemoprevention), given the higher specificity of the new tests as compared to that of the conventional TST. We conducted a systematic review of all publications that have addressed the cost or cost-effectiveness of IGRAs. The objective of this report was to undertake a structured review and critical appraisal of the methods used for the model-based cost-effectiveness analysis of TB screening programmes.

**Methods:**

Using Medline and Embase, 75 publications that contained the terms "IGRA", "tuberculosis" and "cost" were identified. Of these, 13 were original studies on the costs or cost-effectiveness of IGRAs.

**Results:**

The 13 relevant studies come from five low-to-medium TB-incidence countries. Five studies took only the costs of screening into consideration, while eight studies analysed the cost-effectiveness of different screening strategies. Screening was performed in high-risk groups: close contacts, immigrants from high-incidence countries and healthcare workers. Two studies used the T-SPOT.TB as an IGRA and the other studies used the QuantiFERON-TB Gold and/or Gold In-Tube test. All 13 studies observed a decrease in costs when the IGRAs were used. Six studies compared the use of an IGRA as a test to confirm a positive TST (TST/IGRA strategy) to the use of an IGRA-only strategy. In four of these studies, the two-step strategy and in two the IGRA-only strategy was more cost-effective. Assumptions about TST specificity and progression risk after a positive test had the greatest influence on determining which IGRA strategy was more cost-effective.

**Conclusion:**

The available studies on cost-effectiveness provide strong evidence in support of the use of IGRAs in screening risk groups such as HCWs, immigrants from high-incidence countries and close contacts. So far, only two studies provide evidence that the IGRA-only screening strategy is more cost-effective.

## Background

Screening healthcare workers (HCWs) and close contacts for latent tuberculosis infection (LTBI) and active tuberculosis (TB) is fundamental to infection control programmes [1]. For about a century, the tuberculin skin test (TST) has been used to detect LTBI. However, the TST has known limitations, including non-specific reactivity in persons vaccinated with BCG and in those carrying infection with non-tubercular mycobacteria (NTM) [2]. Advances in molecular biology have led to the development of new in-vitro assays that measure the interferon (INF)-γ released by sensitised T cells after stimulation with *M. tuberculosis *antigens. These tests are more specific than the TST, because they use antigens not shared by any of the BCG vaccine strains or by the more common species of NTM (e.g. *M. avium*) [3]. Besides having higher specificity and sensitivity than the TST, IGRAs correlate better with surrogate measures of exposure to *M. tuberculosis *[4-7] and have a higher predictive value for LTBI progression to active TB in close contacts in low-incidence settings [8,9].

There are two different IGRAs available commercially: the ELISA-based QuantiFERON Gold (QFT-G) or QuantiFERON Gold In-Tube (QFT-IT) of Cellestis, Australia, and the T-SPOT-based T-SPOT.TB of Oxford Immunotech, UK. As experience with the IGRAs evolves in routine screening, the IGRAs are endorsed with national recommendations [10-13]. Based purely on financial considerations, it is usually recommended to verify a positive TST with an IGRA and to perform a chest x-ray (CXR) on those who test positive with an IGRA (Nice, DZK and Switzerland).

The first study concerning the cost of introducing the IGRAs in screening for LTBI was the paper by Mori and Harada published in 2005 [14]. As this paper was written in Japanese, only the conclusion is given here: 'It was confirmed that the additional use of QFT would greatly reduce the number of indications for chemoprophylaxis cases that have never been infected and that the use of QFT is cost-effective in spite of its relatively high unit cost.' The QFT-G used for analysis was the second generation of the QuantiFERON-TB. In Europe, this test has long since been replaced by the QuantiFERON-TB Gold In-Tube (QFT-IT). Therefore this study is of rather historical importance, signalling the starting point of analysing the cost-effectiveness of IGRAs in screening for LTBI.

Several cost and cost-effectiveness studies of the introduction of IGRAs in screening for LTBI and treatment of LTBI based on IGRA results have been published in recent years. In this systematic review, the available evidence on the costs and cost-effectiveness of TB screening with IGRAs is analysed.

## Methods

### Search strategy

On 30 June 2010 with an update on 20 Mai 2011, we conducted a Medline and Embase search of articles published. Search terms included 'cost + interferon (IGRA) + tuberculosis'. The searches were limited to studies published in German and English. We identified 76 references. Additional studies were identified from the reference list of articles and relevant reviews. Two of the authors reviewed all of the abstracts and full texts using a review form developed for this purpose. The form contained the following inclusion criteria:

- Study design: cost analysis or cost-effectiveness studies

- Study population: included high-risk groups (HCWs, immigrants, close contacts)

- Outcome: costs, incremental cost ratios

- Screening strategies: TST and/or IGRA

- Languages: English (no study written in German was available)

### Definitions

The following definitions of sensitivity, specificity, negative (NPV) and positive predictive value (PPV) were included into analysis:

Sensitivity is defined as the proportion of persons with true latent TB infection proportion who test positive with the screening test. Specificity is defined as the number of true negatives in screening for latent TB infection divided by the sum of true negatives and false positives. It denotes the ability of a test to assign LTBI-free as test-negative, irrespective of BCG vaccination status.

The NPV of a screening test for latent TB infection is defined as the number of true negative test results divided by the sum of true and false negative results. If sensitivity, specificity and prevalence of LTBI are known in the screened group, the respective formula reads*: specificity × (1-prevalence)/specificity × (1-prevalence) + (1-sensitivity) × prevalence*.

The positive predictive value of the screening test, on the other hand, is the proportion of test-positive persons who are truly infected with *M. tuberculosis*. This can be found as the ratio of: *(sensitivity × prevalence)/sensitivity × prevalence + (1-specifiicty) × (1-prevalence)*

In general, the higher the sensitivity of a screening test and the lower the LTBI prevalence in the test population, the higher the NPV will be. Vice versa, the higher the specificity of a screening test, and the higher the LTBI prevalence, the higher the PPV. Of note, in contrast to specificity of a test for LTBI which can be measured by testing healthy persons without any history of exposure to TB and coming from low burden countries, there is no gold standard against which to establish the sensitivity of a LTBI screening test. The higher the PPV of a test, the lower the number of persons scored positive who will need follow-up examination by CXR, and the lower the number needed to treat in order to prevent a TB case by chemo-prevention and thus the more cost-effective the implementation of such a test.

### Study selection

Studies were included if they used versions of the commercially available IGRAs as a screening test for LTBI and performed any kind of cost or cost-effectiveness analysis based on high-risk populations (HCWs, immigrants and close contacts). The selected studies are described in brief. Special emphasis was given to the assumptions made by the authors about test criteria, the costs of the TST and the IGRAs as well as the assumptions about the probability of progression to active TB in TST or IGRA positives.

Cost ratios for the TSTs and IGRAs in different countries were calculated in order to compare the different costs assumed in the studies without having to take into account the particular currencies of the countries.

The review was carried out in compliance with the prisma-statement http://www.prisma-statement.org

## Results

### Studies identified

**Figure 1:**
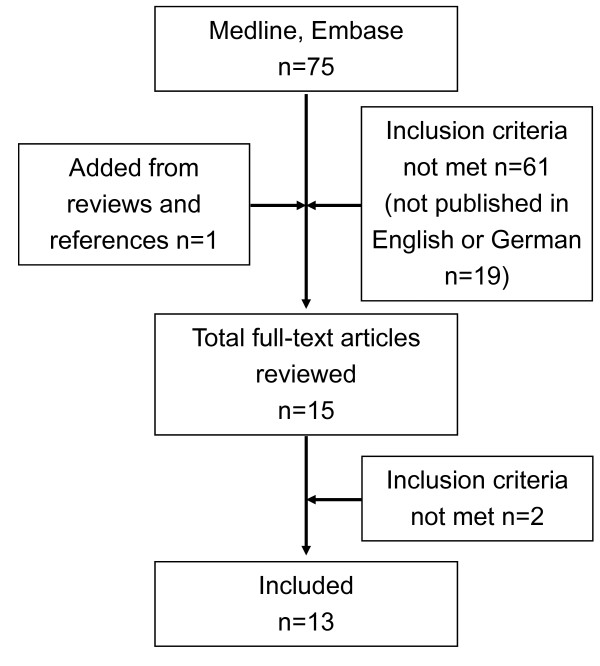
**Flow chart of study selection**.

We identified 75 abstracts from the database search. One abstract was added from references (n = 76). 61 abstracts were excluded because they did not meet the inclusion criteria. Of these excluded studies, one was published in Japanese, but provided an English abstract. Finally, 15 studies were reviewed as full-text articles and 13 articles met the inclusion criteria (Figure 1). One study did not perform a cost-effectiveness analysis of INH treatment based on IGRA results and the other excluded study, which focussed on travellers to high-incidence countries, used TST only. Of the studies included, five contained cost analyses while eight contained cost-effectiveness analyses. A short summary of the strategies used and their basic results are provided in Tables 1 and 2.

**Table 1:** Summary of five cost analysis studies

	Study period	Country	Study population	Outcome	Strategies	Results
Wrighton-Smith et al. [15]	1 year	Switzerland	1000 contacts	Direct cost	TST-only T-SPOT.TB-only TST +T-SPOT.TB	€ 673,245 € 387,135 € 342,563

Fox et al. [16]	Not assessed	Israel	100 HCWs	Costs for screening and treatment	TST-only QFT-only TST+QFT	€ 8,127 € 7,280 € 4,827

Diel et al. [17]	2 years	Germany	Close contacts	Costs	TST-only QFT-only TST+QFT TST+QFT in BCG-vaccinated	€ 91.06 € 61.29 € 52.05 € 55.45

Hardy et al. [18]	Not assessed	UK	Immigrants	Total costs/LTBI identified	QFT-only TST+QFT	₤ 93.16 ₤ 160.81

Diel et al. [19]	2 years	Germany	Close contacts	Costs	TST QFT TST+QFT	€ 232.58 € 215.79 € 227.89

**Table 2:** Summary of eight cost-effectiveness studies

	Study period	Country	Study population	Outcome	Strategies	Results
Pooran et al. [21]	2 years	UK	Close contacts	Incremental costs/active TB case prevented	TST-only T-SPOT.TB-only TST+T-SPOT QFT-only TST+QFT	₤ 47,840 ₤ 39,712 ₤ 37,206 ₤ 42,051 ₤ 37,699

Marra et al. [22]	20 years, 3% discounted	Canada	Close contacts of foreign-born, non-aboriginal Canadian-born and aboriginal TB cases	Incremental costs/QALY	QFT (BCG+) + TST (BCG-) TST+QFT (BCG+) +TST (BCG-) QFT (foreign-born, aboriginal, BCG+) +TST (BCG-) QFT (foreign-born and aboriginal) + TST (others) TST+QFT (foreign-born, aboriginal, BCG+) +TST (others) TST+QFT (foreign-born, ab-original) + TST (others) TST+QFT (all) QFT (all)	Dominant Dominant CAD 31,930 CAD 40,433 CAD 135,672 Dominated Dominated CAD 79,443

Oxlade et al. [23]	20 years, 3% discounted	Canada	Close and casual contacts	Incremental costs/case prevented	No screening TST QFT TST+QFT	TST cost saving in close contacts (and casual contacts from low incidence countries) with exception of contacts receiving BCG after infancy (cost savings for QFT)
			
			Immigrants	Incremental costs/case prevented	No screening CXR only TST only QFT only TST+QFT	least expensive for subjects from high and intermediate incidence countries: CAD 875-30,680 Less expensive than QFT: CAD 46,600 (high incidence) -800,000 (intermediate) Most expensive: CAD 62, 643 (high incidence) 75, 777 (intermediate) Least expensive for subjects from low inci-dence countries): CAD 27,369-45,827

Kowada et al.[24]	Lifetime, 3% discounted	Japan	Close contacts	Incremental costs/QALY	IGRA-only TST-only TST+IGRA	$ 471.54 $ 573.98, dominated by IGRA-only $ 500.55, dominated by IGRA-only

de Perio et al. [25]	Lifetime, 3% discounted	USA	HCWs with no BCG vaccination	Incremental costs/QALY	QFT-IT QFT-G TST	Not assessed $ 14,092 Dominated
					
			HCWs with BCG vaccination		QFT-IT QFT-G TST	Not assessed $ 103,047 Dominated

Deuffic-Burban et al. [26]	Lifetime, 3% discounted	France	Close contacts	Incremental costs/LYG	No screening TST ≥ 10 mm +QFT QFT TST ≥ 5 mm	€ 560 € 730 Strongly dominated

					TST ≥ 5 mm +QFT TST ≥ 10 mm	Weakly dominated Strongly dominated

Diel et al. 2007 [27]	20 years 3% discounted	Germany	Close contacts	Incremental costs/LYG	No treatment TST > 5 TST > 10 QFT TST+QFT	$ 30,170 dominant dominant dominant

Diel et al. [28]	20 years, 3% discounted	Switzerland	Close contacts in middle-aged group	Incremental costs/LYG	TST > 5 mm TST > 10 mm TST > 15 mm T-SPOT-only TST+T-SPOT	€ 141,502 € 107,151 € 44,831 € 23,692 € 23,692
			
			Young group	Incremental costs/LYG	TST > 5 mm TST > 10 mm TST > 15 mm T-SPOT-only TST+T-SPOT	€ 96,705 € 70,955 € 26,451 € 11,621 € 11,621

### Cost-comparison or cost-optimisation studies

Cost-comparison studies analyse the costs of different screening strategies but not the effectiveness of the different treatments.

The Swiss paper analysed the cost of introducing the IGRA in routine screening of contacts and was published in 2006 by Wrighton-Smith and Zellweger [15]. For the analysis, probabilities of a positive TST (> = 10 mm) and a positive IGRA (T-SPOT.TB) were taken from contacts evaluated in 2004 and 2005 in accordance with the Swiss protocol for contact tracing. Only the costs of chemoprevention based on the probability of a positive test were compared while the effect on TB prevention was not taken into consideration. Three strategies were evaluated: IGRA-only, IGRA as a confirmation test for a positive TST, and TST-only. The two-step strategy was less expensive than the IGRA-only strategy, however only by a small margin of 5%. Compared to the TST-only strategy, the costs of the IGRA-only strategy were 44% lower. Compared to the strategy using both tests they were 49% lower.

Fox et al. [16] analysed the costs of screening HCWs for tuberculosis with the IGRA versus the TST. Based on Israeli HCWs, the cost analysis comprised 100 HCWs who were referred for routine screening. TST (> = 10 mm) was positive twice as often as QFT-IT (17% vs. 34%). Assuming a 50% adherence to chemoprevention, the total cost of screening and treating these 100 HCWs was minimised to € 4,155 by using the QFT-IT in order to confirm a positive TST (reduction of 49%). However, the possibility that some HCWs would not come back to get their TSTs read was not included in the model. The probability of having an indeterminate IGRA result was surprisingly high, and the figure provided by the authors was more than 50% in comparison to the probability of having a positive QFT result (0.09 vs. 0.17). Under these circumstances the QFT-IT-only strategy cost 12% less than the TST-only strategy (€ 7,248 vs. € 8,217). Observed adherence to chemoprevention in the QFT-positive group was 47%, compared to 12% in the TST-positive group.

Diel et al. [17] used a decision analytic model to simulate the costs of screening for LTBI in close contacts over a two-year time period. They analysed the costs of the different screening strategies: 1) QFT-only, 2) TST-only, 3) QFT to confirm a positive TST, and 4) positive TST followed by a QFT in BCG-vaccinated contacts. Based on the probabilities of a previous TST/QFT-comparison study among close contacts, the combined TST/QFT strategy was less costly than the TST-only strategy. The cost of the TST-only strategy was 48.6% higher than the QFT-only strategy (€ 91.06 vs. € 61.29 per close contact). The combined screening strategy for all contacts was the least expensive one (€ 52.02 per contact), followed by the strategy which used QFT to confirm a positive TST in contacts with BCG vaccination and for all others TST-only (€ 55.45)

Hardy et al. [18] analysed the screening cost for LTBI in 280 immigrants moving from high-incidence countries to Great Britain based on NICE guidelines or when using the IGRAs first. With few exceptions (pregnancy, young age) all immigrants receive chest x-rays (CXRs) and those from high-incidence countries are also tested with the TST. If the TST is positive, an IGRA is performed. The alternative protocol provides for QFT-IT in all immigrants and stipulates CXRs in those with a positive QFT-IT. The number of chest x-rays needed decreased from 275 to 105 (38%) and the number of QFT-ITs needed increased from 153 to 280 (183%). The number of LTBI cases diagnosed increased from 83 using the NICE protocol to 105 (126%) using the QFT-first protocol. Total costs for the screening of the 280 immigrants were 27% lower for the QFT-first protocol and costs per detected LTBI case were reduced from £ 160.81 to £ 93.16 (-42%). Despite the title ("Cost effectiveness of the NICE guidelines...") no cost-effectiveness analysis was performed.

In a further paper, Diel et al. [19] analysed the health and economic outcomes of isoniazid treatment of 1,000 close contacts followed hypothetically for two years with respect to isoniazid-related hepatotoxicity and early post-exposure TB over a two-year period using the QFT-IT, TST-only or QFT-IT as a test to confirm positive TST results (TST/QFT-IT). The model incorporated the results of a prior predictive value study [8] assuming a higher progression rate to TB disease in QFT-positive than in TST-positive subjects.

Screening and treatment based on QFT-IT-only (€ 215.79 per close contact) was less costly compared to the TST/QFT-IT strategy (€ 227.89) and the TST-only strategy (€ 232.58) because the more targeted preventive therapy provoked fewer secondary hepatotoxic events. There were also fewer missed LTBI cases, due to, among other things, misread or false-negative TST results. This lead to a lower number of unprevented TB cases.

In summary, the IGRA-only screening strategy was less costly than the TST screening strategy in two cost analysis studies [18,19]. Both studies used the QFT-IT. Three other studies found the two-step strategy to be less costly [15-17]. The Swiss study analysed the T-SPOT.TB. In all studies the TST-only strategy was the most expensive one (Table 1).

### Cost-effectiveness studies

Cost-effectiveness studies are done as part of a complete economic evaluation with the aim of comparing the costs and consequences of various measures [20]. All cost-effectiveness studies in this review used Markov modelling for the transition to different health states. This dynamic decision analytic technique allows the progression from LTBI to active TB and the treatment outcome to be modelled over time. The follow-up period after MTB infection varied from two years to life-long in the various studies and all studies discounted the costs and health effects using a rate of 3%. Most of these studies used 'quality-adjusted life years' (QALYs) and 'life years gained' (LYGs) as effects and calculated incremental cost effectiveness ratios (ICERs). One study, Pooran et al. [21], provided cost per avoided TB cases instead. A strategy is considered dominant if it is less expensive and at the same time more effective than the alternative strategy, which then becomes the dominated strategy (Table 2).

Pooran et al. [21] analysed the cost-effectiveness of five different screening scenarios in contact tracing over a two-year time period in the UK using: 1) TST-only, 2) the T-SPOT.TB-only, 3) positive TST followed by T-SPOT.TB, 4) QFT-IT-only, and 5) positive TST followed by QFT-IT.

Cost-effectiveness was measured as total costs per active TB case and the ICER per active TB case prevented. Cost for performing the T-SPOT were assumed to be only ₤ 55. Figures provided for sensitivity and specificity of the T-SPOT were clearly higher than those for the QFT (95% and 100% vs. 89% and 95%); sensitivity for the TST was assumed to be 85%. There was no stratification with respect to BCG vaccination, and TST specificity was considered to be 80% for all contacts. The total cost of TST screening amounted to ₤ 199,598 per 1,000 contacts compared to T-SPOT.TB at ₤ 203,983, QFT-IT at ₤ 202,921, TST/T-SPOT at ₤ 162,387 and TST/QFT-IT at ₤ 157,048. The incremental cost per active TB case prevented, compared with no screening, was ₤ 47,840 in TST, ₤ 39.712 in T-SPOT.TB, ₤ 42,051 in QFT. The most cost-effective strategy was the two-step strategy with TST and T-SPOT (₤ 37,206), followed very closely by the TST/QFT strategy (₤ 37,699).

To assess the cost-effectiveness of QFT-G vs. the TST in diagnosing contact persons with active TB cases in Canada, Marra et al. [22] used a decision analytic Markov model. Three different screening strategies were evaluated over a 20-year-period: TST-only, QFT-only and the two-step strategy using the QFT-G to confirm a positive TST. The model was stratified by ethnicity (foreign-born, non-aboriginal Canadian-born and aboriginal), and BCG vaccination status, as the noted groups have different rates of prior infection and BCG use.

The most cost-effective strategy was to administer QFT-G in BCG-vaccinated contacts and reserve TST for all other patients, assuming specificity for the TST of more than 99% in all BCG-unvaccinated subgroups, but of only 96% for the QFT. Driven primarily by the extremely high specificity value, which was not varied in sensitivity analysis, and in combination with an assumed low BCG vaccination rate, the TST alone-strategy is dominant (ICER) and an incremental net monetary benefit (INMB) of CA$ 3.70 per contact investigation was calculated. The least cost-effective strategy was the use of the QFT-G for all cases, which resulted in an INMB of CA$ -11.15.

Oxlade et al. [23] used Markov modelling to compare expected TB cases and costs over 20 years following screening for TB with different strategies among hypothetical cohorts of foreign-born immigrants and close contacts in Canada. Canada. The authors compared five different strategies for immigrants: 1) no screening, 2) CXR, 3) TST-only, 4) QFT-only, and 5) QFT for confirmation of a positive TST. For screening of contact persons they compared three strategies: 1) no screening, 2) TST only and 3) QFT.

The least costly strategy for immigrants coming from intermediate and high incidence countries versus non screening was CXR screening. There, patients were considered positive for LTBI if they had an "abnormal" radiograph and subsequently a positive TST. The sensitivity of that method for identifying those infected was assumed to be 11%. The approach had an incremental cost of CA$ 825 and $ 30,680 per prevented case when applied to entry screening of immigrants from high-incidence and intermediate incidence countries, respectively. In contrast, initial screening with QFT was the most expensive one when immigrants had no BCG vaccination or had been BCG vaccinated in infancy.

With respect to contact screening, assuming a TST specificity of 98% in BCG-unvaccinated cases, of 92% among infants and 60% among older BCG-vaccinated, screening of close contacts and casual contacts (when coming from low-incidence countries) with QFT or TST would result in savings compared to non-screening. In such circumstances, TST would generally be more cost-effective than the QFT with the exception of screening older close and casual contacts who had received BCG vaccination. However, Kowada et al. [24] evaluated the cost-effectiveness of the QFT-IT for TB screening in close contacts in Japan over the lifetime of a contact (age 20) in a nearly completely BCG vaccinated society. They compared the QFT-only strategy with the TST followed by the QFT strategy and TST-only strategy. The target population was a hypothetical cohort of 1,000 immunocompetent 20-year-old close contacts to a sputum smear positive index case. Based on a very low baseline specificity of the TST among BCG vaccines of 15% and a high prevalence of LTBI, the QFT-only strategy was dominant (US$ 471.54/28.1099 QALYs) compared to the TST/QFT strategy (US$ 500.55/28.1087 QALYs) and the TST-alone strategy (US$ 573.98/28.1079 QALYs). The incremental cost-effectiveness ratio of the QFT was a cost saving of US$23.043/QALYs. Accordingly, the QFT-only strategy is the most cost-effective for contact investigation in a medium-incidence country like Japan.

In addition, de Perio et al. [25] used a Markov state transition decision model to compare cost and quality-adjusted life years (QALYs) with three strategies for a hypothetical 35-year-old HCW cohort with and without BCG vaccination, also over a lifetime horizon. Costs and QALYs were discounted at 3% per year. They compared two versions of QFT and TST-only and accounted for inadequate and indeterminate outcomes of both QFTs, for failure to return for TST reading, and for 2-step TST testing. In this study, sensitivity of the QFT was assumed to be clearly higher than that of the TST (76% vs. 67%) and the drop-out rate for reading of the first TST was high at 12%. Both IGRAs were more effective and less costly than the TST. The TST strategy was thus dominated. The ICER of the QFT-G compared with the QFT-IT was US$14.092/QALY for BCG-unvaccinated and US$103.047/QALY for BCG-vaccinated HCWs. In conclusion, the authors stated that the use of IGRAs leads to superior clinical outcomes and lower costs than screening with the TST does.

In contrast to these findings, four other cost-effectiveness studies showed that the two-step strategy with TST and IGRA was the most cost-effective strategy compared to the IGRA-only strategy. The first one is a cost-effectiveness study among adult close contacts in France. Deuffic-Burban [26] generated a decision analytic model. Lifetime costs and life expectancies for no testing, TST (basically positive at a cut off of ≥ 10 mm) and QFT-IT only and TST/QFT-IT strategies were calculated and compared using incremental cost-effectiveness ratios (ICERs) in euros per life year gained (LYG). The authors provided a sensitivity figure of only 76% for the QFT (vs. 73% for the TST) and a very high cost to cost relation between the QFT (€ 40.50) and the TST (€ 2.16). The proportion for adherence to LTBI treatment as a basic value for their calculations was assumed to be only 57%, thus reducing the cost for preventative INH treatment initially started due to false positive TST results. Given these assumptions, the discounted direct medical lifetime costs of care per patient were € 417 for no testing, € 476 for TST, € 443 for QFT and € 435 for TST/QFT. The TST/QFT strategy was associated with an ICER of € 560/LYG compared to no testing, while the QFT-only strategy was associated with an ICER of € 730/LYG. The TST-only strategy, irrespective of whether a cut off of 5 or 10 mm was used, was strongly dominated (higher costs and lower life expectancy).

Another study from Diel et al. [27] assessed the cost-effectiveness of the QFT assay for screening and treatment of close contacts in Germany. They analysed the health and economic outcomes of isoniazid treatment for 20-year-old contacts over a 20-year time period using two different cut-off values for the TST (≥ 5 and 10 mm), the QFT-only and then QFT as a confirmatory test for a positive TST. QFT-based treatment led to cost savings of US$ 542.9 and 3.8 life days per LTBI case compared to non-treatment, TST-based treatment at 10 mm induration size saved US$ 177.4 and saved 2.0 life days per test-positive contact. Choosing a 5 mm cut-off for the TST resulted in additional expenditures and saved only 0.9 days. Although the ICER for treatment based on a TST < 5 mm was below the commonly used willingness-to-pay threshold (US$ 30,170/LYG) it resulted in unnecessary treatment of 77% due to false-positive TST results. Combining TST at a 5 mm cut-off followed by the QFT in a dual-step screening approach was only marginally less expensive (0.6%) than using the QFT solely.

In a further study, Diel et al. [28] analysed the outcomes of INH treatment of close contacts in Switzerland using the Markov model over a 20-year period following screening with the TST-only (three different cut-off values: 5, 10 and 15 mm) and the T-SPOT.TB-only or as a two-step strategy with the TST. T-SPOT.TB-based treatment was cost-effective in both age groups at € 11,621 (20-yr-old cohort) and € 23,692 per LYG (40-yr-old cohort). Only in the younger group, and with a TST cut-off of > 15 mm, was the ICER of € 26,451/LY below the willingness-to-pay threshold of US$ 50,000 (or € 40,195; average exchange rate for 2004: US$ = € 0.8039); all other TST-only options were not cost-effective. Combination of the TST with T-SPOT.TB slightly reduced the total cost compared with the T-SPOT.TB alone, by 4.4% and 5.0% in the younger and older groups respectively.

The ultimate aim of LTBI screening is the prevention of progression to active TB via chemopreventative therapy. Whether the introduction of IGRA in the TB screening of contacts is cost-effective (in terms of producing expenditures below a predefined WTP threshold per LYG) in this respect was analysed in eight studies from six different countries (US, Canada, Japan, Switzerland, France, UK and Germany). With the exception of Japan, a country with medium TB prevalence, these countries are considered to have low TB prevalence. All studies performed TB screening on groups at high risk for developing tuberculosis: HCWs [25], close contacts [15,19,21,22,24,26-28], and immigrants from high-incidence countries [23].

### Screening strategies

One study analysed the alternative use of TST or IGRA [25] and seven studies compared the 1) TST-only, 2) positive TST followed by IGRA, and 3) IGRA-only strategies [21-24,26-28].

One study used the T-SPOT.TB [28], one study used both IGRAs [21] and all the others used a version of the QFT as IGRA. The QFT-G [22-24,26] was used in four studies, one study used QFT-G as well as QFT-IT [25] and three studies used QFT-IT [19,21,27].

### Assumptions

#### a) Specificity and sensibility

Assumptions on TST specificity ranged from as low as 15% in Japanese contacts with repeated BCG vaccination [24] to 99% in non-vaccinated Canadian contacts [22] and from 95% up to 100% for the IGRAs [21,25]. Based on TST-positive/IGRA-negative discordant results in the source populations, specificity of the TST was assumed to be 34% in Swiss contacts [28] and 67% in German contacts [19], regardless of vaccination status (Table 2). Most studies assumed for TST and IGRA an equal or at least similar sensitivity for the diagnosis of LTBI (Table 3) ranging from 75-99% for the TST and from 76-100% for IGRAs. If sensitivity differed between the TST and any IGRA in the same study it was in favour of the IGRA by a margin of 1-9%.

**Table 3:** Assumed specificity and sensitivity of TST and IGRA in cost-effectiveness studies depending on different countries

Country	Specificity			Sensitivity	
	**TST/BCG**		**QFT/** **(T-SPOT)**		

	**No**	**Yes**		**TST**	**QFT/** **(T-SPOT)**

Canada 2007 [23]	98%	60%	98%	95%	95%

Canada 2008 [22]	99%	62%	96%	99%	99%

Japan 2008 [24]	98%	≥ 15%*	96%	71%	76%

USA 2009 [25]	98%	70%	100%	67%	76%

Germany 2007 [27]	90%	60%	100%	90%	90%

Switzerland 2007 [28]	34%	34%	-	88%	95%

UK 2010 [21]	-	80%	95%	85%	89% (100%)

France 2010 [26]	-	60%	96%	73%	76%

* Depending on repeated BCG in adolescence and adulthood

#### b) Costs

Because of the different currencies used in the studies, the costs of the TST and IGRA cannot be compared directly. Therefore the ratio "cost for IGRA divided by cost for TST" was calculated (Table 4). This ratio was highest for the study using the T-SPOT.TB (5.6). For the QFT studies this ratio ranged from 1.18 to 4.6.

**Table 4:** Costs for TST and IGRA depending on different countries

Country	Currency	TST	IGRA	Ratio	Difference
Canada 2007 [23]	CAD	12.73	19.0	1.49	6.23

Canada 2008 [22]	CAD	25.41	45.32	1.78	20.32

Japan 2008 [24]	$	7.4	34.2	4.6	25.8

USA 2009 [25]	$	12.48	31.18	2.5	8.7

Germany 2007 [27] *	$	145.99	171.78	1.18	25.79

Germany 2009 [19] **	€	117.5	145.98	1.24	27.48

Switzerland 2007 [28]	€	23	129	5.6	106

UK 2010 [21]	₤	15.433	55.00/45.00	3.6/2.9	39.6/29.6

France 2010 [26]	€	10.86	44.83	4.1	33.97

* CXR and consultation if test is positive are included

** Study includes salary of staff

#### c) Progression rates

Whilst figures for sensitivity and specificity of the TST and IGRA varied considerably, in all but one study [19] progression rates to active TB were assumed to be comparable for TST and IGRA. Based on literature searches, progression rates were subdivided depending on diameter in TST in four out of the seven studies [23,24,27,28]. A fixed lifetime progression rate of 0.01 was used in the US study [25]. In one Canadian study [23] the progression rate used was available neither from the text nor from the author (e-mail request). In the other [22], an age-dependant rate of between 0.0055 (age 20-30) and 0.0018 (age 40-50) per year was used. Annual risk of progression was assumed to be 0.0037-0.0056 for the T-SPOT.TB [27] and 0.0030-0.0056 for the QFT [28]. Based on a recent progression study [8,9] rates were assumed to be 0.0228 for TST positives and 0.1463 for QFT positives for a two-year period [19]. In Deuffic-Burban [26], progression rates were calculated separately following each additional year following recent infection (8.66% in the first, declining to 0.24% per year after ≥ 5 years following LTBI). In Pooran et al. [21], 2.5% for the two years post-exposure was assumed. Kowada et al. [24] provided a baseline figure of annual 0.37% per Markov cycle.

#### d) Results of different screening strategies

Regardless of the differences of the assumed progression rates for active TB and the different costs for the TST and IGRA, in all studies the TST-only strategy was the most expensive one. The study of de Perio [21] supported an IGRA-only strategy vs. the TST first, IGRA-second strategy, assuming a clearly higher sensitivity of IGRA than TST given a nearly identical specificity for the QFT-IT (100%) and the TST (98%), but did not assess a dual-step strategy.

In four out of seven available studies the dual-step strategy (IGRA following TST positive subjects) was the least expensive strategy and in two studies [19,24] the IGRA-only strategy was less expensive. In those two studies, the most striking difference was found in the German analysis that determined the IGRA-only strategy for contacts to be the most cost-effective, applying different progression rates for TST and IGRA based on observed progression rates in Germany [8]. In close contacts followed for two years the progression rate in QFT-IT positives was 6.4 times higher than in close contacts with TST ≥ 5 mm. Kowada's study [24] assumed an only slightly better sensitivity of the QFT (76% vs. 71% for the TST) but - as stated above - an extraordinarily high difference in specificity, which led to the favourable outcome for the QFT-only strategy.

Clearly, with the exception of de Perio's work [25], the difference in outcomes of the compared strategies does not result from the sensitivity assumptions made for TST and IGRA by the various authors. Noteworthy, in fact, is that lacking or only small differences in the sensitivities assumed [21,24,26,28], now made questionable by developments in the literature [29], tipped the balance in favour of the dual-step strategy and against the IGRA-only strategy.

Given that the two studies that favoured the IGRA-only strategy were valid in respect of the progression rates and the specificity of the TST, assumptions about these two factors appear to be decisive for the outcome of the cost-effectiveness analysis.

## Discussion

Decision-making in healthcare is increasingly based on cost-effectiveness considerations. This trend is reflected in most national recommendations concerning TB screening (e.g. NICE [11], Germany [12], Switzerland [13]). These recommendations favour a dual-step testing strategy (TST first, IGRAs second in TST-positive subjects) because in the cost-effectiveness studies available at the time, the possibility of there being substantially different rates of sensitivity and specificity between the methods being compared was not taken into consideration. Increased knowledge about progression rates in those with a positive IGRA helps to improve the outcome modelling of hypothetical cohorts used for cost-effectiveness analysis. The ultimate aim of LTBI screening is the prevention of progression to active TB via chemoprevention. Whether the introduction of IGRA in the TB screening of contacts is cost-effective (in terms of producing expenditures below a predefined WTP threshold per LYG) in this respect was analysed in eight studies from six different countries (US, Canada, Japan, Switzerland, France, UK and Germany). With the exception of Japan, a country with medium TB prevalence, these countries are considered to have low TB prevalence. All studies performed TB screening on high risk groups for developing tuberculosis: HCWs [25], close contacts [15,19,22,24,26-28], or immigrants from high-incidence countries [23]. However, given the lack of consistency in the critical assumptions (e.g. different assumptions on the test parameters, especially of specificity for both test systems, where assumptions varied between 34 and 98% even among non-BCG vaccinated for the TST and between 95% and 100% for the IGRAs, it has to be pointed out that the studies considered in that review cannot be directly compared to each other. This is also due to the extremely different cost ratio between TST and IGRA assumed; it varied between 1.2 and 5.6.

So far, the assumption of a different progression rate in direct comparison of TST-positive and IGRA-positive subjects is based on two publications [8,9]. In a latter abstract, reported at the annual meeting of the BTS in 2009, progression rates for QFT positive contacts were 17.2% in two years; these rates were even higher than those assumed in the German studies (14.6% and 12.9%, respectively).

Different assumptions on sensitivity of the TST and IGRA did not explain why two studies favoured the IGRA-only strategy, whereas four studies found the IGRA in TST positives to be the most cost-effective. However, a recent meta-analysis of TST and IGRA sensitivity for active TB indicates that the sensitivity assumed for the TST and IGRA was quite high and that the differences between sensitivity of TST and IGRA might be as large as 14%, well outside of the 1% to 9% range assumed in the papers reviewed here [30]. If the higher sensitivity of the IGRA is confirmed, this would reduce the costs for the treatment of undetected active TB and LTBI that progresses to active TB. This would give further support to the IGRA-only strategy.

With respect to the costs for test performance, differences occurred not only due to different currencies but also because some studies used the manufacturer's costs needed to perform the tests and other studies also took the costs for manpower into consideration [19], or combined costs for testing with costs for CXRs, chemoprevention [27] and costs for hepatitis developed during IHN treatment [25].

Comparison of the studies is hampered not only by the different costs assumed and different assumptions on test parameters used in the studies but also by different strategies in modelling and different outcomes used. Only three of the eight cost-effectiveness studies used 'quality-adjusted life years' (QALYs) as outcome measures [22,24,25], two [21,23] used averted TB cases and three used 'life years gained' (LYGs) [26-28]. With evolving methodology in cost-effectiveness studies it would be reasonable to use the same outcomes (QALYs) in order to increase the comparability of the different studies.

Most of the studies published so far were performed in low-to-medium-incidence, high-income countries. It remains to be analysed whether their findings can be confirmed in high-incidence countries.

## Conclusion

The available studies on cost-effectiveness provide strong evidence in support of the use of IGRAs in screening high-risk groups, such as HCWs, immigrants from high-incidence countries, and close contacts. In general, the higher unit cost of the IGRAs compared to that of the TST is compensated for by cost savings through the more targeted performance of CXRs and offering of chemoprevention. If the increasing evidence that IGRA positive subjects have a higher probability of progression to active TB holds true, the IGRA-only screening strategy should prove to be the more cost-effective test. However, until the body of research in this area is broadened to include generally accepted inputs for economic analysis, recommendations concerning this matter should be regarded with caution.

## Competing interests

RD has received reimbursement for attending scientific conferences and/or fees for speaking from Cellestis and Oxford Immunotec. All other authors declare that they have no competing interests.

## Authors' contributions

RD, JTC and AS have made substantial contributions to the conception of the article and have been involved in revising the manuscript critically for important intellectual content. They have given final approval of the version to be published. AN has made substantial contributions to the conception and design of the review. He has been involved in drafting the manuscript and has given final approval of the version to be published.

## Pre-publication history

The pre-publication history for this paper can be accessed here:

http://www.biomedcentral.com/1472-6963/11/247/prepub
